# Viral Discovery and Sequence Recovery Using DNA Microarrays

**DOI:** 10.1371/journal.pbio.0000002

**Published:** 2003-11-17

**Authors:** David Wang, Anatoly Urisman, Yu-Tsueng Liu, Michael Springer, Thomas G Ksiazek, Dean D Erdman, Elaine R Mardis, Matthew Hickenbotham, Vincent Magrini, James Eldred, J. Phillipe Latreille, Richard K Wilson, Don Ganem, Joseph L DeRisi

**Affiliations:** **1**Department of Biochemistry and BiophysicsUniversity of California San FranciscoSan Francisco, CaliforniaUnited States of America; **2**Department of Microbiology and ImmunologyUniversity of California San FranciscoSan Francisco, CaliforniaUnited States of America; **3**National Center for Infectious DiseasesCenters for Disease Control and PreventionAtlanta, GeorgiaUnited States of America; **4**Department of Genetics, Genome Sequencing CenterWashington University School of MedicineSt. Louis, MissouriUnited States of America

## Abstract

Because of the constant threat posed by emerging infectious diseases and the limitations of existing approaches used to identify new pathogens, there is a great demand for new technological methods for viral discovery. We describe herein a DNA microarray-based platform for novel virus identification and characterization. Central to this approach was a DNA microarray designed to detect a wide range of known viruses as well as novel members of existing viral families; this microarray contained the most highly conserved 70mer sequences from every fully sequenced reference viral genome in GenBank. During an outbreak of severe acute respiratory syndrome (SARS) in March 2003, hybridization to this microarray revealed the presence of a previously uncharacterized coronavirus in a viral isolate cultivated from a SARS patient. To further characterize this new virus, approximately 1 kb of the unknown virus genome was cloned by physically recovering viral sequences hybridized to individual array elements. Sequencing of these fragments confirmed that the virus was indeed a new member of the coronavirus family. This combination of array hybridization followed by direct viral sequence recovery should prove to be a general strategy for the rapid identification and characterization of novel viruses and emerging infectious disease.

## Abstract 2

Know your enemy. Description of the ‘virus gene chip’ that helped to classify the SARS virus as a novel coronavirus

## Introduction

Over the past two decades, technological advances in molecular biology have fuelled progress in the discovery of new pathogens associated with human diseases. The identification of novel viruses such as hepatitis C virus (Choo et al. 1989), sin nombre virus (Nichol et al. 1993), and Kaposi's sarcoma herpesvirus (Chang et al. 1994) has relied upon a diverse range of modern molecular methods such as immunoscreening of cDNA libraries, degenerate PCR, and representational difference analysis, respectively. In spite of these successes, there remain numerous syndromes with suspected infectious etiologies that continue to escape identification efforts, in part due to limitations of existing methodologies for viral discovery (Muerhoff et al. 1997; Kellam 1998). These limitations, coupled with the constant threat posed by newly emerging infectious diseases of unknown origin, necessitate that new approaches be developed to augment the repertoire of available tools for pathogen discovery.

We have previously described a prototype DNA microarray designed for highly parallel viral detection with the potential to detect novel members of known viral families (Wang et al. 2002). This microarray contained approximately 1600 oligonucleotides representing 140 viruses. Building upon this foundation, a more comprehensive second-generation DNA microarray consisting of 70mer oligonucleotides derived from every fully sequenced reference viral genome in GenBank (as of August 15, 2002) was constructed. The most highly conserved 70mers from each virus were selected as described by Wang et al. (2002) to maximize the probability of detecting unknown and unsequenced members of existing families by cross-hybridization to these array elements. On average, ten 70mers were selected for each virus, totaling approximately 10,000 oligonucleotides from approximately 1,000 viruses. The objective was to create a microarray with the capability of detecting the widest possible range of both known and unknown viruses. This pan-viral microarray was used as part of the global effort to identify a novel virus associated with severe acute respiratory syndrome (SARS) in March 2003, as reported by Ksiazek et al. (2003). We describe here the experimental details of the microarray methodology for novel virus identification, using the SARS outbreak as an example.

## Results

**Figure 1:**
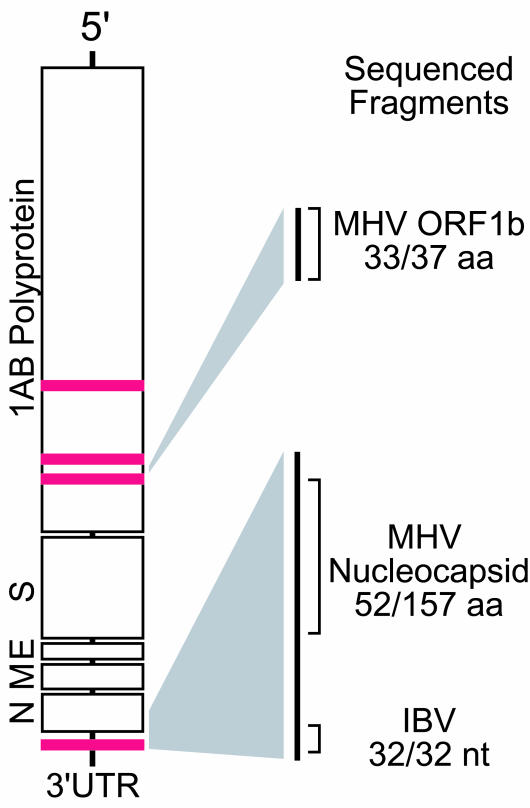
Prototypical Coronavirus Genome Structure Red bars indicate physical location of virus microarray DNA elements mapped to a generic coronavirus genome. Portions of the coronavirus genome sequenced by physical recovery and PCR methods are highlighted with homologies to known coronaviruses. Abbreviations: aa, amino acid; nt, nucleotide

During the initial phase of research into the etiology of SARS, an unknown virus was cultured in Vero cells from a patient suffering from SARS (Ksiazek et al. 2003). Total nucleic acid purified from this viral culture, as well as a control culture, was obtained from the Centers for Disease Control and Prevention on March 22, 2003. These two samples, along with additional controls (HeLa cell RNA and water alone), were amplified and hybridized within 24 h to the virus DNA microarray. The strongest hybridizing array elements from the infected culture were derived from two families: astroviridae and coronaviridae. Table 1 lists the oligonucleotides from these families with the greatest hybridization intensity. By comparison, these oligonucleotides yielded essentially background levels of hybridization in the various control arrays performed in parallel. The initial suggestion from this hybridization pattern was that members of both of these viral families might be present. However, alignment of the oligonucleotides using ClustalX revealed that all four hybridizing oligonucleotides from the astroviridae and one oligonucleotide from avian infectious bronchitis virus (IBV) (GenBank NC_001451), an avian coronavirus, shared a core consensus motif spanning 33 nucleotides (data not shown); thus, these five oligonucleotides behaved essentially as multiple redundant probes for the same sequence. This motif is known to be present in the 3′ UTR of all astroviruses and the avian coronaviruses (Jonassen et al. 1998), but appears to be absent in the available sequenced mammalian coronaviruses (bovine coronavirus, murine hepatitis virus [MHV], human coronavirus 229E, porcine epidemic diarrhea virus, and transmissible gastroenteritis virus). The other three hybridizing oligonucleotides were derived from three conserved regions within the ORF1AB polyprotein common to all coronaviruses (Figure 1). Based on the aggregate hybridization pattern, the virus appeared to be a novel member of the coronavirus family.

**Table 1:**
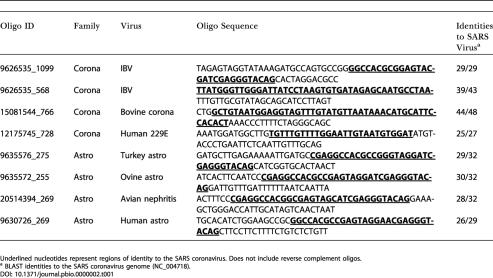
Oligonucleotides Hybridizing to Viral Sample

Underlined nucleotides represent regions of identity to the SARS coronavirus. Does not include reverse complement oligos

^a^ BLAST identities to the SARS coronavirus genome (NC_004718)

To further characterize this virus, we sequenced fragments of the viral genome using two complementary approaches. First, BLAST alignment of two of the hybridizing viral oligonucleotides, one each from bovine coronavirus and human coronavirus 229E, to the IBV genome indicated that the oligonucleotides possessed homology to distinct conserved regions within the *NSP11* gene (BLAST identity matches of 42/47 and 26/27, respectively). A pair of PCR primers was designed to amplify the intervening sequences between the two conserved regions, and a fragment that possessed 89% identity over 37 amino acids to MHV, a murine coronavirus, was obtained (Figure 1; sequence available as Data S1).

**Figure 2:**
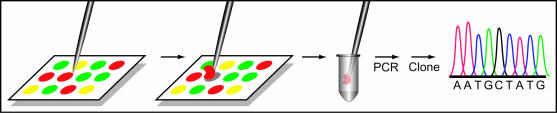
Viral DNA Recovery and Sequencing Scheme Hybridized viral sequences were physically scraped from a DNA microarray spot, amplified, cloned, and subsequently sequenced.

In a parallel approach, we directly recovered hybridized viral sequences from the surface of the microarray. This procedure took advantage of the physical separation achieved during microarray hybridization, which effectively purified the viral nucleic acid from other nucleic acid species present in the sample**.** Using a tungsten needle, the DNA microarray spot corresponding to the conserved 3′ UTR motif was repeatedly scraped and the hybridized nucleic acid was recovered. This material was subsequently amplified, cloned, and sequenced (Figure 2). The largest clone spanned almost 1.1 kb; this fragment encompassed the 3′ UTR conserved motif and extended into the most 3′ coding region of the viral genome. BLAST analysis revealed 33% identity over 157 amino acids to MHV nucleocapsid, thus confirming the presence of a novel coronavirus (see Figure 1; see Data S1). We subsequently confirmed results obtained from both strategies described above by using a random-primed RT-PCR shotgun sequencing approach that generated contigs totaling approximately 25 kb of viral genome sequence (see Data S1).

## Discussion

In this report, we have demonstrated the viability of detecting novel pathogens via cross-hybridization to highly conserved sequence motifs. With the recent sequencing of the complete SARS coronavirus genome (GenBank NC_004718) (Marra et al. 2003; Rota et al. 2003), we were able to retrospectively determine the degree of nucleotide identity shared between the hybridizing oligonucleotides and the new coronavirus genome (see Table 1). Stretches of relatively uninterrupted nucleotide identity as short as 25 nucleotides yielded clearly detectable hybridization signal, confirming that novel viruses with only limited homology to known viruses can be successfully detected by this strategy.

A key feature of this approach is that direct recovery of hybridized material from the microarray provides a rapid route for obtaining sequences of novel viruses. By contrast, conventional strategies for subsequent sequence identification would require time-consuming steps such as library screening or additional rounds of PCR primer design and synthesis. In the case of SARS, we were able to ascertain within 24 h that a novel coronavirus was present in the unknown sample, and partial genome sequences of this virus were obtained over the next few days without the need for specific primer design. To our knowledge, this is the first demonstration of the feasibility and utility of directly recovering nucleic acid sequences from a hybridized DNA microarray. In light of the continuous threat of emerging infectious diseases, this overall approach will greatly facilitate the rapid identification and characterization of novel viruses.

## Materials and Methods

### 

#### Nucleic acid isolation

Total nucleic acid was purified using the automated NucliSens extraction system (BioMerieux, Durham, North Carolina). Following the manufacturer's instructions, 100 μl of each specimen was added to tubes containing 900 μl of prewarmed NucliSens lysis buffer and incubated at 37°C for 30 min with intermittent mixing. Fifty microliters of silica suspension provided in the extraction kit was added to each tube and mixed. The mixtures were then transferred to a nucleic acid extraction cartridge and loaded onto the extractor workstation for processing. Approximately 50 μl of total nucleic acid eluate was recovered.

#### Amplification

For the culture supernatants, 450 ng of nucleic acid was used as input for the amplification protocol. In parallel, 50 ng of HeLa cell RNA was used as a positive amplification control and water was used for a negative control. Samples were amplified using a random-primer protocol as described by Wang et al. (2002), with the following modifications: first- and second-strand synthesis were primed using primer-A (5′-GTTTCCCAGTCACGATCNNNNNNNNN) followed by PCR amplification using primer-B (5′-GTTTCCCAGTCACGATC) for 40 cycles. Aminoallyl-dUTP was incorporated into the PCR product using an additional 20 cycles of thermocycling. A detailed protocol is available as Protocol S1.

#### Microarray hybridization and analysis

DNA microarrays were printed and hybridized essentially as described by Wang et al. (2002), with the following modifications: for array printing, a single-defined 70mer (spike-70) was mixed with each viral oligonucleotide in a 1:50 ratio. Array hybridizations used Cy5-labeled amplified probe from either virally infected cultures or controls (mock-infected culture, HeLa RNA, or water); a reference signal for every spot on each array was generated by using a Cy3-labeled version of the reverse complement of spike-70. Oligonucleotides were assessed by Cy5 intensity. Oligonucleotides from the astrovirus and coronavirus families that passed a conservative, arbitrarily set cutoff of (Cy5_infection_-Cy5_mock_) > 1500 intensity units are listed in Table 1. Additional oligonucleotides from these families and their homology to the SARS coronavirus are listed in Table S1. Array data has been deposited in the Gene Expression Omnibus (GEO) database (accession number GSE546). A complete list of the viral oligonucleotide sequences on the microarray is also available as Table S2.

#### Conventional PCR using array element sequences

PCR primers were designed by aligning the hybridizing oligonucleotides (Oligo IDs 15081544_766 and 12175745_728) to the IBV genome (Fwd: 5′-TGTTTTGGAATTGTAATGTGGAT; Rev: 5′-TACAAACTACCTCCATTACAGCC) and selecting stretches of near-identity. Primer-B-amplified material was used as the template for 35 cycles of thermocycling using the following program: 94°C for 30 s, 56°C for 30 s, and 72°C for 60 s.

#### Direct sequence recovery from the microarray

Amplified viral sequences hybridized to individual microarray spots were recovered by scraping a 100 μm area of the microarray using a tungsten wire probe (Omega Engineering, Inc.) mounted on a micromanipulator while visualized by fluorescence microscopy (Nikon TE300). Recovered material was PCR amplified using primer-B, cloned into pCR2.1TOPO (Invitrogen), and sequenced. A detailed protocol is available as Protocol S2.

#### Shotgun sequencing

Primer-B-amplified nucleic acid (see above) was cloned in pCR2.1TOPO, plated on 2xYT/kan plates, and grown overnight at 37°C. White colonies were picked into 384-well plates containing 2xYT/kan plus 8% glycerol and incubated overnight at 37°C. DNA was purified by magnetic bead isolation. DNA sequencing involved adding 3 μl of water to each bead pellet, followed by 3 μl of Big Dye terminator (v3.1) sequencing cocktail, and incubation for 35 cycles of 95°C for 5 s, 50°C for 5 s, and 60°C for 2 min. Reaction products were ethanol precipitated, resuspended in 25 μl of water, and loaded onto the ABI 3730xl sequencer. The resulting sequence reads were trimmed to remove primer sequences from the RT-PCR step and then assembled by Phrap (P. Green, unpublished data). Resulting contigs were screened by blast to remove any contigs with high human or monkey sequence similarity. The remaining contigs were edited to high quality, making any obvious joins. (Sequences are available as Data S1.)

## Supporting Information

Data S1 Supporting Data (91.5 KB DOC)

Protocol S1 Supporting Protocol (28 KB DOC)

Protocol S2 Supporting Protocol (39.5 KB DOC)

Table S1 Supporting Table (97 KB DOC)

Table S2 Supporting Table (2.2 MB XLS)

### Accession Numbers

The Gene Expression Omnibus accession number for the array sequence is GSE546.

## Abbreviations

IBV: avian infectious bronchitis virus
MHV: murine hepatitis virus
SARS: severe acute respiratory syndrome
