# Supplementary material for: Viral Discovery and Sequence Recovery Using DNA Microarrays
Source: PLoS Biol. 2003 Nov 17;1(2):e2. doi: 10.1371/journal.pbio.0000002 (PMC261870; doi:10.1371/journal.pbio.0000002)
Supplement: Data S1 — (91.5 KB DOC) [file pbio.0000002.sd001.doc]

Conventional PCR fragment

>Coronavirus nsp11 fragment

ATTTCGCCCTTTACAAACTACCTCCATTACAGCCTGGTAAGTTCAAGTTTGACAAGACTCTTGTGTCAAACCTACACACAATTGCATTGGCTGGGTAACGATCCACATTACAATTCCAAAACAAAGGG

Array Recovery Cloned Fragment

>Coronavirus nucleocapsid and 3' UTR fragment

CAATGGTCGAGCTGGATCCNNTAGTAAACGCCCGCCAGGTGTGGCTGGAATTCCCCCCCTTGTTTTCCCAGTCACGATAGCGGAGGTGGTGAAACTGCCCTCGCGCTATTGCTGCTAGACAGATCGAACCAGCTTGAGAGCAAAGTTTCTGGTAAAGGCCAACAACAACAAGGCCAAACTGTCACTAAGAAATCTGCTGCTGAGGCATCTAAAAAGCCTCGCCAAAAACGTACTGCCACAAAACAGTACAACGTCACTCAAGCATTTGGGAGACGTGGTCCAGAACAAACCCAAGGAAATTTCGGGGACCAAGACCTAACCAGACAAGGAACTGATTACAAACATTGGCCGCAAATTGCACAATTTGCTCCAAGTGCCTCTGCATTCTTTGGAATGTCACGCATTGGCATGGAAGTCACACCTTCGGGAACATGGCTGACTTATCATGGAGCCATTAAATTGGATGACAAAGATCCACAATTCAAAGACAACGTCATACTGCTGAACAAGCACATTGACGCATACAAAACATTCCCACCGACAGAGCCTAAAAAGGACAAAAAGAAAAAGACTGATGAAGCTCAGCCTTTGCCGCAGAGACAAAAGAAGCAGCCCACTGTGACTCTTCTTCCTGCGGCTGACATGGATGATTTCTCCAGACAACTTCAAAATTCCATGAGTGGAGCTTCTGCTGATTCAACTCAGGCATAAACACTCATGATGACCACACAAGGCAGATGGGCTATGTAAACGTTTTCGCAATTCCGTTTACGATACATAGTCTACTCTTGTGCAGAATGAATTCTCGTAACTAAACAGCACAAGTAGGTTTAGTTGACTTTAATCTCACATAGCAATCTTTAATCAATGTGTAACATTAGGGAGGACTTGAAAGAGCCACCACATTTTCATCGAGGCCACGCGGAGTACGATCGAGGGTACAGTGAATAACGCTAGGGAGAGCTGCCTATATGGAAGAGCCCTAATGTGTAAAATTAATTTTAGTAGTGCTACCCCCTATCGTGACTGGGAAACAAGGGCGAATTCNGCNNAGATATNCCA

Shotgun Sequencing Contigs

>Contig112

GTCGGCTTAGGAAATCCAAAGGCTATCAAGTGTGTGCCTCAGGCTGAAGT

AGAATGGAAGTTCTCCGATGCTCAGCCATGTAGTGACAAAGCTTACAAAA

TAGAGGAGCTCTTCTATTCTTATGCTACACATCACGATAAATTCACTGAT

GGTGTTTGTTTGTTTTGGAATTGTAACGTTGATCGTTACCCAGCCAATGC

AATTGTGTGTAGGTTTGACACAAGAGTCTTGTCAAACTTGAACTTACCAG

GCTGTGATGGTGGTAGTTTGTATGTGAATAAGCATGCATTCCACACTCCA

GCTTTCGATAAAAGTGCATTTACTAATTTAAAGCAATTGCCTTTCTTTTA

CTATTCTGATAGTCCTTGTGAGTCTCATGGCAAACAAGTAGTGTCGGATA

TTGATTATGTTCCACTTTTTTCTGCTACGTGTATTACACGATGCAATTTA

GGTGGTGCTGTTTGCAGACACCATGCAAATGAGTACCGACAGTACTTGGA

TGCATATAATATGATGATTTCTGCTGGATTTAGCCTATGGATTTACAAAC

AATTTGATACTTATAACCTGTGGAATACATTTACCAGGTTACAGAGTTTA

GAAAATGTGGCTTATAATGTTGTTAATAAAGGACACTTTGATGGACACGC

CGTA

>Contig113

TACTACCAGCTATCGTGAAGCAGCTTGCTGCCACTTAGCAAAGGCTCTAA

ATGACTTTAGCAACTCAGGTGCTGATGTTCTCTACCAACCACCACAGACA

TCAATCACTTCTGCTGTTCTGCAGAGTGGTTTTAGGAAAATGACATTCCC

GTCAGGCAAAGTTGAAGGGTGCATGGTACAAGTAACCTGTGGAACTACAA

CTCTTAATGGATTGTGGTTGGATGACACAGTATACTGTCCAAGACATGTC

ATTTGCACAGCAGAAGACATGCTTAATCCTAACTATGAAGATCTGCTCAT

TCGCAAATCCAACCATAGCTTTCTTGTTCAGGCTGGCAATGTTCAACTTC

GTGTTATTGGCCATTCTATGCAAAATTGTCTGCTTAGGCTTAAAGTTGAT

ACTTCTAACCCTAAGACACCCAAGTATAAATTTGTCCGTATCCAACCTGG

TCAAACATTTTCAGTTCTAGCATGCTACAATGGTTCACCATCTGGTGTTT

ATCAGTGTGCCATGAGACCTAATCATACCATTAAAGGTTCTTTCCTTAAT

GGATCATGTGGTAGTGTTGGTTTTAACATTGATTATGATTGCGTGTCTTT

CTGCTATATGCATCATATGGAGCTTCCAACAGGAGTACACGCTGGTACTG

ACTTAGAAGGTAAATTCTATGGTCCATTTGTTGACAGACAAACTGCACAG

GCTGCAGGTACAGACACAACCATAACATTAAATGTTTTGGCATGGCTGTA

TGCTGCTGTTATCAATGGTGATAGGTGGTTTCTTAATAGATTCACCACTA

CTTTGAATGACTTTAACCTTGTGGCGGCGTA

>Contig114

TAGCTGGTTTATCCAGATTGTTAACGATTACTTGGTTGGCATTAATACAG

CCACCATCGTAACAATCAAAGTATTTATCAACAACTTCAACTACGAATAG

GAGTTGTCTGATATCACACATTGTTGGCAGATTATAACGATAATAGTCAT

AATCACTGATAGCAGCGTTGCCATCCTGAGCAAAGAAGAAGTGTTTTAGT

TCAACAGAACTTCCTTCCTTAAAGAAACCTTTAGACACAGCAAAGTCATA

AAAGTCTTTATTAAAATTACCGGGTTTGACAGTTTGAAAAGCAACATTGT

TTGTTAGTGCAGCTACTGAAAAGCATGTAGTGCGTTTATCTAGCAATAAA

TTGCCAGAAGCTGCATGCATAGCTGGATCAGCAGCATACACTAAAAGTTC

CTTGAAACTGAGACGCGAGCTATGTAAGTTTACATCCTGATTATGTACGA

CTCCTAACTCACGAAAATGGTATCCAGTTGAAACAACAAAAGGAACACCA

TCTACAAATATTTTTCTTACTAGTGGTCCAAAACTTGTAGGTGGAAACAC

AGTAGAAAATAGCACATTAAAGTTTGCACAATGAAGGATACACCTATCAT

CCAAACAGTTAATACAATTGGGATGGTATGTCTGGTCCCAATATTTAAAA

TAACGGTCGAAGAGACAAAGTCTCTCTTCCGTAAAATCATATTTCAGCAA

ATCCCACTTAATAAGTGGTTTTGCGAGATCAGCATCCATATGGGACTCAG

CAGCCAATGCCCTAGTCAAAGTGAGGATGGGCATCAGCAATGAGTAATAT

GAATCCACAATAGGAACTCCGCAGCCTGGTGCTACTATCGTGAAAGGGAA

ACAAGGGCGAATTCTGC

>Contig117

TACATCACTGACTAAAGGCACACTAGAACCAGAATATTTTAATTCAGTGT

GCAGACTTATGAAAACAATAGGTCCAGACATGTTCCTTGGAACTTGTCGC

CGTTGTCCTGCTGAAATTGTTGACACTGTGAGTGCTTTAGTTTATGACAA

TAAGCTAAAAGCACACAAGGATAAGTCAGCTCAATGCTTCAAAATGTTCT

ACAAAGGTGTTATTACACATGATGTTTCATCTGCAATCAACAGACCTCAA

ATAGGCGTTGTAAGAGAATTTCTTACACGCAATCCTGCTTGGAGAAAAGC

TGTTTTTATCTCACCTTATAATTCACAGAACGCTGTAGCTTCAAAAATCT

TAGGATTGCCTACGCAGACTGTTGATTCATCACAGGGTTCTGAATATGAC

TATGTCATATTCACACAAACTACTGAAACAGCACACTCTTGTAATGTCAA

CCGCTTCAATGTGGCTATCACAAGGGCAAAAATTGGCATTTTGTGCATAA

TGTCTGATAGAGATCTTTATGACAAACTGCAATTTACAAGTCTAGAAACA

CCACGTCGCAATGTGGCTACATTACAAGCAGAAAATGTAACTGGACTTTT

TAAGGACTGTAGTAAGATCATTACTGGTCTTCATCCTACACAGGCACCTA

CAAACCTCAGCGTTGATATAAAGTTCAAGACTGAAGGATTATGTGTTGAC

ATACCAGGCATGC

>Contig120

AGAATTCGCCCTTGTTTCCTATTCACGATACTTGGCCTTTTCTGTTTACT

CAACCGTTACTTCAGGCTTACTCTTGGTGTTTATGACTACTTGGTCTCTA

CACAAGAATTTAGGTATATGAACTCCCAGGGGCTTTTGCCTCCTAAGAGT

AGTATTGATGCTTTCAAGCTTAACATTAAGTTGTTGGGTATTGGAGGTAA

ACCATGTATCAAGGTTGCTACTGTACAGTCTAAAATGTCTGACGTAAAGT

GCACATCTGTGGTACTGCTCTCGGTTCTTCAACAACTTAGAGTAGAGTCA

TCTTCTAAATTGTGGGCACAATGTGTACAACTCCACAATGATATTCTTCT

TGCAAAAGACACAACTGAAGCTTTCGAGAAGATGGTTTCTCTTTTGTCTG

TTTTGCTATCCATGCAGGGTGCTGTAGACATTAATAGGTTGTGCGAGGAA

ATGCTCGATAACCGTGCTACTCTTCAGGCTATTGCTTCAGAATTTAGTTC

TTTACCATCATATGCCGCTTATGCCACTGCCCAGGAGGCCTATGAGCAGG

CTGTAGCTAATGGTGATTCTGAAGTCGTTCTCAAAAAGTTAAAGAAATCT

TTGAATGTGGCTAAATCTGAGTTTGACCGTGATGCTGCCATGCAACGCAA

GTTGGAAAAGATGGCAGATCAGGCTATGACCCAAATGTACAAACAGGGAA

GATCTGAGGACAAGAGGGCAACTTA

>Contig135

GTCCACCATGAGAAATGGCCCATAAGCTTGTAAAGGTCAGCATTCCAAGA

ATGCTCTGTTATCTTTACAGCTATAGAACCACCCAGGGCTAGTTTTTGCT

TTATAAATCCACACAGATAAGTGAAAAACCCTTCTTTAGAGTCATTCTCT

TTTGTCACATGTTTGGTCCTAGGGTCATACATATCGCTAATAATAAGGTC

CCATTTATTAGCCGTATGTACTGTTGCACAGTCTCCAATTAAAGTAGAAT

CTGCGTCGGAGACGAAGTCATTAAGATCTGAATCGACAAGTAGTGTGCCA

GTTGGCAACCATTGTCTGAGCACAGCTGTACCTGGTGCAACTCCTTTATC

AGAGCCAGCACCAAAGTGAATAACTCTCATGTTGTAGGGTACAGCTAAAG

TAAGTGTATTTAAGTATTGACACAGTTGAGTATACTTTGCGACATTCATC

ATTATTCCTTTTGGTATAACAGCATTTTCACCATAATTCTGAAGGTCACA

CTTTTCAAGAAGCATTCTTTGCATCTTGTACAAGTTAGGCATCGCAACAC

CTGGTTGCCACGCTTGACTTGCTTGTAGTTTTGGGTAGAAGGTTTCAACA

TGTCCATCCTTACACCAAAGCATGAATGAAATTTCAGCATAGTCAATTGT

AACCTTGACCACTTTTGAAATCACTGACAAATCTTGTGACTTTATTATCT

CGACAAAGTCATCAAGTAAAAGATCAATCACAGAACACACACATTTTGAT

GAACCTGTTTGCGCATCTGTTATGAAGTAATTTTTCACTGTGCTGTCCAT

AGGGATAAAATCCTCTAATTTAAGTGGTGAATCTTGTGAGCGCTTGGCTA

AGCCTATCATTAAATGAAGACCGCCAAGTTGTCCATGACTGAAATCTCCA

TAAACGATGTGTTCGAAGGCATAGCCCTCGAGCTTATATCGCTGTATGAA

TTCATCCATAGCGAGCTCGAGAAAGTCAGTTTCCATTTGTGATCTGGGCT

TAAAATCCTCTAAGTCTCTGCTCTGAGTAAAGTAGGTTTCAGGCAACTGT

TGAATAATGCCGTCTACTTTCTTAAAGTAGTTAAACTGTGTTTTTACTGA

TTCTCCAATTAATGTGACTCCATTGACGCTAGCTTGTGCTGGTCCCTTTG

AAGGTGTTAGACCTTTGACTGAACCTTCTGTTATTAAAACACCATTACGG

GCGTTTCTAAAAAGGTCTACCTGTCCTTCCACTCTACCATCAAACAAGAC

AGTAAGTGAAGAACAAGCACTCTCAGTAGGTTTCTTGGCAATGTCAGTCA

TTGTGCAGACACCTATTGTAGATGCATGTGCTGGGGCTTCTCTTTTGTAG

TCCCAGATTACAGTATTAGCAGCGATATCAACACCCAAATTATTGAGTAC

CTTAATCTCTGGCACCGGTTTAATGTTACGCTTAGCCCAAAGCTCAAATG

CAACATTAACAGGAAGTGTTGTCTTATTTTCAAAGATCTCCACATCAATA

CCATCTACCTTTGTGTAAACAGCATTATTAATGATGGAAACAGGTGCTTC

GCCGGCGTGTCCATCAAAGTGTCCTTTA

>Contig195

AACGATAAGCTGGTTTCCCATTTAATAAATGGGGTAAGGCTAGACTTTAT

TATGACTCAATGAGTTATGAGGATCAAGATGCACTTTTCGCGTATACTAA

GCGTAATGTCATCCCTACTATAACTCAAATGAATCTTAAGTATGCCATTA

GTGCAAAGAATAGAGCTCGCACCGTAGCTGGTGTCTCTATCTGTAGTACT

ATGACAAATAGACAGTTTCATCAGAAATTATTGAAGTCAATAGCCGCCAC

TAGAGGAGCTACTGTGGTAATTGGAACAAGCAAGTTTTACGGTGGCTGGC

ATAATATGTTAAAAACTGTTTACAGTGATGTAGAAACTCCACACCTTATG

GGTTGGGATTATCCAAAATGTGACAGAGCCATGCCTAACATGCTTAGGAT

GATGGCCTCTCTTGTTCTTGCTCGCAAACATAACACTTGCTGTAACTTAT

CACACCGTTTCTACAGGTTAGCTAACGAGTGTGCGCAAGTATTAAGTGAG

ATGGTCATGTGTGGCGGCTCACTATATGTTAAACCAGGTGGAACATCATC

CGGTGATGCTACAACTGCTTATGCTAATAGTGTCTTTAACATTTGTCAAG

CTGTTACAGCCAATGTAAATGCACTTCTTTCAACTGATGGTAATAAGATA

GCTGACAAGTATGTCCGCAATCTACAACACAGGCTCTATGAGTGTCTCTA

TAGAAATAGGGATGTTGATCATGAATTCGTGGATGAGTTTTACGCTTACC

TGCGTAAACATTTCTCCATGATGATTCTTTCTGATGATGCCGTTGTGTGC

TATAACAGTAACTATGCGGCTCAAGGTTTAGTAGCTAGCATTAAGAACTT

TAAGGCAGTTCTTTATTATCAAAATAATGTGTTCATGTCTGAGGCAAAAT

GTTGGACTGAGACTGACCTTACTAAAGGACCTCACGAATTTTGCTCACAG

CATACAATGCTAGTTAAACAAGGAGATGATTACGTGTACCTGCCTTACCC

AGATCCATCAAGAATATTAGGCGCAGGCTGTTTTGTCGATGATATTGTCA

AAACAGATGGTACACTTATGATTGAAAGGTTCGTGTCACTGGCTATTGAT

GCTTACCCACTTACAAAACATCCTAATCAGGAGTATGCTGATGTCTTTCA

CTTGTATTTACAATACATTAGAAAGTTACATGATGAGCTTACTGGCCACA

TGTTGGACATGTATTCCGTAATGCTAACTAATGATAACACCTCACGGTAC

TGGGAACCTGAGTTTTATGAGGCTATGTACACACCACATACAGTCTTGCA

GGCTGTAGGTGCTTGTGTATTGTGCAATTCACAGACTTCACTTCGTTGCG

GTGCCTGTATTAGGAGACCATTCCTATGTTGCAAGTGCTGCTATGACCAT

GTCATTTCAACATCACACAAATTAGTGTTGTCTGTTAATCCCTATGTTTG

CAATGCCCCAGGTTGTGATGTCACTGATGTGACACAACTGTATCTAGGAG

GTATGAGCTATTATTGCAAGTCACATAAGCCTCCCATTAGTTTTCCATTA

TGTGCTAATGGTCAGGTTTTTGGTTTATACAAAAACACATGTGTAGGCAG

TGACAATGTCACTGACTTCAATGCGATAGCAACATGTGATTGGACTAATG

CTGGCGATTACATACTTGCCAACACTTGTACTGAGAGACTCAAGCTTTTC

GCAGCAGAAACGCTCAAAGCCACTGAGGAAACATTTAAGCTGTCATATGG

TATTGCTACTGTACGCGAAGTACTCTCTGACAGAGAATTGCATCTTTCAT

GGGAGGTTGGAAAACCTAGACCACCATTGAACAGAAACTATGTCTTTACT

GGTTACCGTGTAACTAAAAATAGTAAAGTACAGATTGGAGAGTACACCTT

TGAAAAAGGTGACTATGGTGATGCTGTTGTGTACAGAGGTACTACGACAT

ACAAGTTGAATGTTGGTGATTACTTTGTGTTGACATCTCACACTGTAATG

CCACTTAGTGCACCTACTCTAGTGCCACAAGAGCACTATGTGAGAATTAC

TGGCTTGTACCCAACACTCAACATCTCAGATGAGTTTTCTAGCAATGTTG

CAAATTATCAAAAGGTCGGCATGCAAAAGTACTCTACACTCCAAGGACCA

CCTGGTACTGGTAAGAGTCATTTTGCCATCGGACTTGCTCTCTATTACCC

ATCTGCTCGCATAGTGTATACGGCATGCTCTCATGCAGCTGTTGATGCCC

TATGTGAAAAGGCATTAAAATATTTGCCCATAGATAAATGTAGTAGAATC

ATACCTGCGCGTGCGCGCGTAGAGTGTTTTGATAAATTCAAAGTGAATTC

AACACTAGAACAGTATGTTTTCTGCACTGTAAATGCATTGCCAGAAACAA

CTGCTGACATTGTAGTCTTTGATGAAATCTCTATGGCTACTAATTATGAC

TTGAGTGTTGTCAATGCTAGACTTCGTGCAAAACACTACGTCTATATTGG

CGATCCTGCTCAATTACCAGCCCCCAGTATCTGGGTGXXXXXXXXXXXXX

XXXXXXX

>Contig203

GGTAGTATCTAAGGCTCCACTGAAATACTTGTACTTGTTATATAGAGCAA

GATACCTGTTATACTGTGTAAGTGGCAACAGTGTCTCGCTACGCAATTTT

AGGTACATTTCCTTGTTGAGCAAAAAGGTACACAAAGCAGCCTCCTCGAA

GGTACTAAATGTAACTCCATTAAACATGACTCTTTTCCTAAGATAGTTGT

TAAAGAACCAATGGCAGTGCTTCAGAGAAATACAGAATACATAGATTGCT

GTTATCCAAAAAGGCACAATAGGAGAAAACATGGCAAACCATTGAAGGTG

AGCCAAGAATGAAACATCATTGGTGAAATAGAATGTCAAGTACAAGTAAA

AGACTGAGTAGACTCCCGGCAGAAAGCTGTAAGCTGGTACCAGACAGAGT

ATAGTGAAAGACATCAAAAACAAAAGTGCATTAGCAGCAACAACATGGTT

GTACTCACCAAAAACACGTCTGAATTTCATAAAGTAGTAGGCAGCACAAG

TCACCAATATGGCAATAATACCACCAGCCACTACTGAAGCAGACACATCT

AAAGCACCCACAGGTTGCACAAGAGGAGTAAAGATGTTAGCTATGAGATT

CATCGCATCAACACCACAGAAAACTCCTGATAGAGCTCTGTAATGCTCAT

TATTAAGAACCCATCTACCACTGGTAGATAGGCAAATACCTACTTCTGAC

CTTTCGCATGTACCATGTCTACAGTACTCAGCATCAAAAGTTGTTACTAC

TCTAACAGAACCCTCCAGGTAAGTGTTAGGAAACTGTATGATGGAACCAT

CCATAAGCACATAACGAGTGTCTGGACGAAGCTCACTATAAGAAATAGAA

CCCTCTAGCAAATTAGTGTCATAACAATATGGCACAGGTTTGCCCATAGC

ATCCTTAAAAATTGTACACTCAGCAGCAAGAACGCAAGCAGAGGTAGCAA

AATCACTATACTCAATGAGTTTGGAAGGTGTGTAGCAAATGTTGCCAACA

GCACTAAAAACACGAGGTAGAAAATGCAAGAAGTCACCATTGATTGCTCT

CAGCACAGTACCCGGTAAGCCAGGCACTATGAAACCAATCTCTCTTGTAA

TGATAGCAGCTACTACAGGGCAGCTTTTGTCATTTTTGTATGAACCACCA

CGCTGGCTAAACCATGCGTCAAAACCAGCATGTTTATTTGCAAAACAGTC

ATCAGTAGAAATGATGTCACGAGTGACACCATCCTGAATGGCTTTGTAAC

CAATGATTTCATTTGTGTAACCATCATGGATTGACAATGTATGTACTGGC

ATAACGATATAACAAACCAATGCAGCAAGAACGCACAATAATGTGGCCTT

AAGCATAAGTTTAAAACAAGTACTAACAATCTTACCACCCTTGAGTGAGA

TTTTAGTAGTTATGACATTGACAACCTGTCTAGTTGTAGCACAAGTTAGT

CTAAAAGGTATGTTGTTCTTCTTGGCAGCACTACGAATTTGTTTACGCAG

CTGTTCAGATAAAGACATGTAGTCTTTTACATTCCAGATGAGTGAAACAT

TGTGACTTTTTGCTACTTGGGCATTGATATGCCTTGCATTACAGTCAATA

CATGCACCAAGATCTCTGGGCGCCATGTTTTCAACCTTATTATAGGTGAG

CATGAAATTGTTACAACTGTCACCTGTCACTTCTAAGTCAGAGTGATGTG

AAAGTTTGAGACATTCAATAACATCCTTTGTGTCAACATCGGTATCAACA

ACACCTTGTCGGGCAGCTGACACGAATGTAGAAAGGACACCATCTAAAGC

TACACCCTTTGCTAACTCGCTGTGAGCTGTAGCAACAAGTGCCTTAAGTT

TTTCCATAGGAACACTAAAAGTTGCTGAAAAGGTGTCGACATAAGCATCA

AACATCTTAACGGAAACTTCAGTACTATCTCCAACGTCTGATACAAGAAC

TTGGTCAAGCAACAGAATAGGTTGGCACATCAGCTGACTGTAGTACACAG

AAGCAGACTTAGAAGCAGACTCGTCGCATTTGGACTTGCCATCAAAAACT

ATGACATTAATAGGCAGTGAACCTTTAGTGTTGTTAGCTCTCAAATTGTC

TAAATTGACAAAATGGGAGAGCGGATGTCTCTCATAGGTCTTTTGACCAG

CCTTGTCAAAGTAGAGGTGAAGCGCGCCATTTTTCACAGCAACACTACCA

ACAATA

>Contig207

TGTTGCACTATTTCGCGCTTGATTCCCACCTTTAATAGCATCTCCGACTG

CCTTTCTCCACCACATCTCTGTTTCCCACCCACTGTAGGTGTTGCCCGTT

CTCTAGATCGGTTCCCGAGTCACGAGACGTCCCACTGTATGACGCCCTTC

TTTATCACCTTTCTTGAAACTCGCTGAATAGTGTCTATAGTCAATAGCCA

CTACATCGCCATTCAAGTCTGGGAAGAATGTGACAGATAGCTCTCGTGAA

GCTGGCTTTGTGAAGCCTGTCATTTGATTTAAATCATCAGCAAATTTTGT

GTTAGAACATGTGAGTTTGAAATTATCAAAACTCGCATTTGGTAATGGTT

GAGTTGGTACAAGGTCTATAGGCTGCTCTGTATAGTAAGCATTATCCTTT

TTATAATACCCATCCAATTTTGGTTCAATCTCTGTGTAAGTAACTCCATC

GAGTTTATACGACACAGGCTTGATGGTTGTAGTGTAAGATGTTTCCTTGT

AGAAAACATCAGTCACTGGTCCTTTGTACTCTGACATCTTTGTAAGGTGA

GCTCCGTCAATACGATAGAGGGTCTCCTTAGCAGTTATATGAGTGTAATG

ACCACACTGATAGTTACCAGTGTACTCATTCGCACATAAGAATGTACCTT

GCTGTAATTTATACTCAGCAGGTGGTGCAGACATCATAACAAAAGAAGAC

TCTTGTTGTACTAGATATTGTGTAGCATCACGACCACACACACATGGAAT

GGAAACACCTGTCTTAAGATTATCATAAGATAGAGTACCCATATACATCA

CAGCTTCTACACCCGTTAAGGTAGTAGTTTTCTGACCACAATGTTTACAC

ACCACATTAAGAACTCGCTTTGCAGATTCCAAATTAGCATGCTGTAGAAG

ATGGGTCATAGTTTCTCTGACATCACCAAGCTCGCCAACAGTTTTATTAC

TGTAAGCGAGTATGAGTGCACAAAAGTTAGCAGCATCACCAGCACGGGCT

CTATAATAAGCCTCTTGAAGTGCTGGTGCATTGAATTTGACTTCAAGCTG

TTGAAGTGCTAATAAAACACTAGACAAATAACAATTGTTATCAGCCCATT

TAATTGAAGTTAAACCACCAACTTGAGGAAATTTCCATTTCTTTGTGTGG

TTTAAAGCAGACATGTACCTACCAAGAAAACTCTCATCAAGAGTATGGTA

GTACTCGAAAGCTTCACTACGTAGTGTGTCATCACTAGGTAGTACAAAGA

AAGTCTTACCCTCATGATTTACATGAGGTTTAATTTTTGTAACATCAGCA

CCATCCAAGTATGTTGGACCAAACTGCTGTCCATATGTCATAGACATATC

CACAAGCTGTGTGTGGAGATTAGTGTTGTCCACAGTTGTGAACACTTTTA

TAGTCTTAACCTCCCGCAGGGATAAGAGACTCTTTAGTTTGTCAAGTGAA

AGAACCTCACCGTCAAGATGAAACTCGACGGGGCTCTCCAGAGTGTGGTA

CACAATTTTGTCACCACGCTTAAGAAATTCAACACCTAACTCTGTACGCT

GTCCTGAATAGGACCAATCTCTGTAAGAGCCAGCCAAAGAAACTGTTTCT

ACAAAGTGCTCCTCAGATGTCTTTGATGACGAAGTGAGGTATCCATTATA

TGTAGTAACAGCATCTGGTGATGATACTGACACTACGGCAGGAACTTTAA

GAAGGCATCGTATGGGTTGCAACTGAGGGAGCCTTGAATACACCCCCTAT

CGTGACT

>Contig225

NNNNNNNNNNNNNNNNNNNNNNNNNNNGCTACGAAGTCATACCAATCCTT

CTTATTGAAATAATCATCATCACAGCAATTGTATGTGACGAGTATTTCTT

TTAATGTATCACAATTACCCTCATCAAAATGACGTAGAGCATAGACTAAA

TCAGCCATTGTGTATTTAGTTAGACGCTGACGTGATATATGTGGTACCAT

GTCACCATCTACTCTAAACTTGAAAAAGTCATGGACAGCAACCGCTGGAC

AATCTTTAACCAAGTTATAAATAGTCTCTTCATGTTGGTAGTTAGACATA

GTATGCCTCTTAACTACAAAGTAAGAGTCTAATAAATTGCCTTCCTCATC

CTTCTCCTGGAAGCGACAGCAATTAGTTTTTAGGAACTTTGCAAAACCAG

CAACTTTTTCGTTGTAAATATCAAAAGCCCTGTAGACGACATCAGTACTA

GTGCCTGTGCCGCACGGTGTAAGACGGGCTGCACTTACACCGCAAACCCG

TTTAAAAACGTTGATGCATCCGCAGACTGCATCAAGGGTTCGCGGAGTTG

GTCACAACTACAGCCATAACCTTTCCACATTCCGCAGACGGTACAGACTG

TGTTTCTAAGTGTAAAACCCACTGGGTCATTAGCACAAGTGGTAGGTATT

TGGACGTACTTACCTTTCAAGTCACAGAATCCTTTAGGATTTGGATGGTC

AATGTGGCATCTACAATACAGACAACATGAAGCACCACCAAAGGACTCTT

GGTCCATGTTAGCTTCTGGTGTTACAGTAATTGCCTGTCCTGTACCAGTG

TGTGTACACAACATCTTCACACAGTTGGTGATTGGTTGTCCTCCACTTGC

TAGGTAATCCTTATATGCTTTAGCAGGGTCTACTGCAAAAGCACAGAAGG

AAAGCACAGTTGAATTGGCAGGTACTTCTGTAGCATTTCCAGCCTGAAGA

CGTACTGTAGCAGCTAAACTGCCCAGCACCATACCTCTATTTAGGTTGTT

TAAGCCTTTGATGAAGTACAAGTATTTCACTTTAGGCCCTTTTGGTGTGT

CTGTAACAAACCTACAAGGTGGTTCCAGTTCTGTGTAAATTGTACCTGTA

CCATCACTCTTAGGGAATCTAGCCCATTTGAGATCTTGGTGGTCTGATAG

TAATGCCAGCACAAACCTACCTCCCTTCGAATTGTTATAGTAGGCAAGTG

CATTGTCATCAGTACAAGCTGTTTGTGTGGTACCAGCCGCACAGGACATC

TGTCGTAGTGCTACTGGACTCAGTTCATTATTCTGTAGTTTAACAGCTGA

GTTGGATCTTAGAGCTGTAACAATAAGAGGCCAAGCCAAATTTGGTGAAT

TGTCCATGTTAATTTCACTAAGTTGAACAATCTTGCTATCCGCATCTTTT

CAAAGTGCGTANNNNNNNNNNNN

>Contig227

CAGAATTCGCCCTTGTTTCCCTCCATGGAAGGAAGATGAGTGATTCATTT

AAATTTTTAGCGACCTCATTGAGGCGGTCAATTTCTTTTTGAATGTTGAC

GACAGAAGCGTTAATGCCTGAAATGTCGCCAAGATCAACATCTGGTGATG

TATGATTTTTGAAGTACTTGTCCAGCTCTTCTTTGAATGAGTCGAGCTCA

GGTTGCAGAGGATCATAAACTGTGTTGTTAATGATGCCAATAACGACATC

ACAATTTCCTGAGACAAATGTATTGTCTGTAGTAATTATTTGTGGAGAAA

AGAAGTTCCTCTGTGTAATAAACCAAGAAGCGCCATCAAACACAAAAACT

CCTTCACGAGGGAAGTATGCTTTGCCTTCATGACAAATTGCTGGCGCTGT

GGTGAAGTTCCTCTCCTGGGATGGCACATACCTGACATGTAGGAAGACAA

CACCATGCGGGGCTGCTTGTGGGAAGGACATAAGGTGGTAGCCCTTTCCA

CAAAAGTCAACTCTTTTTGATTGTCCAAGAACACACTCAGACATTTTAGT

AGCAGCAAGATTAGCAGAAGCCCTGATTTCAGCAGCCCTGATTAGTTGTT

GTGTTACATAGGTTTGAAGGCTTTGAAGTCTGCCTGTAATTAACCTGTCA

ATTTGTACCTCCGCCTCGACTTTATCAAGTCGCGAAAGGATATCATTTAG

CACACTTGAAATTGCACCAAAATTAGAGCTAAGTTGTTTAACAAGTGTGT

TTAATGCTTGAGCATTCTGGTTAACAACGTCTTGCAGCTTGCCCAATGCA

GTTGATGTTGTTGTAAGTGATTCTTGAATTTGACTAATCGCCTTGTTAAA

TTGGTTGGCGATTTGTTTTTGGTTCTCATAGAGAACATTTTGGGTAACTC

CAATGCCATTGAACCTATATGCCATTTGCATAGCAAAAGGTATTTGAAGA

GCAGCGCCAGCACCAAATGTCCATCCAGCAGTGGCAGTACCACTAAATAG

AGCAGCAGTGTAGGCAGCAATCATATCATCAGTGAGCAGAGGTGGCAACA

CTGTAAGTCCATTGAACTTCTGCGCACAAATGAGATCTCTAGCATTAATA

TCACCTAGGCATTCGCCATATTGCTTCATGAAGCCAGCATCAGCGAGTGT

CACCTTATTAAAGAGCAAGTCCTCAATAAAAGACCTCTTAGTTGGCTTTA

GAGGGTCAGGTAATATTTGTGAAAAATTAAAACCACCAAAATATTTCAAA

GTTGGGGTTTTGTACATTTGTTTGACTTGAGCGAACACTTCACGTGTGTT

GCGATCCTGTTCAGCAGCAATACCTGAGAGTGCACGATTTAGTTGTGTGC

AAAAGCTACCATATTGGAGAAGCAAATTAGCACATTCAGTAGAATCTCCG

CAGATGTACATATTACAATCTACGGAGGTTTTAGCCATAGAAACAGGCAT

TACTTCTGTAGTAATGCTAATTGAAAAGTTAGTAGGTATAGCAATGGTGT

TATTAGAGTAAGCAATTGAACTATCAGCACCTAAAGACATAGTATAAGCC

ACAATAGATTTTTGGCTAGTACTACGTAATAAAGAAACTGTATGGTAACT

AGCACAAATGCCAGCTCCAATAGGAATGTCGCACTCATAAGAAGTGTCGA

CATGCTCAGCTCCTATAAGACAGCCTGCTTGAGTCTGGAATACATTGTTT

CCAGTAGAATATATGCGCCAAGCTGGTGTGAGTTGATCTGCATGAATTGC

TGTAGAAACATCAGTGCAGTTAACATCTTGATATAGAACAGCAACTTCAG

ATGAAGCATTTGTTCCAGGTGTAATTACACTTACACCCCCAAAAGAGCAA

GGTGAAATGTCTAATATTTCAGATGTTTTAGGATCTCGAACGGAATCAGT

GAAATCAGAAACATCACGGCCAAATTGTTGAAATGGTTGAAATCTCTTTG

AAGAAGGAGTTAACACACCAGTACCAGTGAGTCCATTAAAATTAAAATTG

ACACACTGGTTCTTAATAAGGTCAGTGGATAATTTTGGTCCACAAACCGT

GGCCGGTGCATTTAAAAGTTCAAAAGAAAGTACTACAACTCTGTAAGGTT

GGTAGCCAATGCCAGTAGTGGTGTAAAAACCATAATCATTTAATGGCCAA

TAACAATTAAGAGCAGGTGGGGTGCAAGGTTTGCCATCAGGGGAGAAAGG

CACATTAGATATGTCTCTCTCAAAGGGCCTAAGCTTGCCATGTCTAAGAT

ACCTATATTTATAATTATAATTACCAGTTGAAGTAGCATCAATGTTCCTA

GTATTCCAAGCAAGGACACAACCCATGAAATCATCTGGCAATTTATAATT

ATAATCAGCAATAACACCAGTTTGTCCTGGCGCTATTTGTCTTACATCAT

CTCCCTTGACTACAAAAGAATCTGCATAGACATTGGAGAAGCAAAGATCA

TTCAACTTAGTGGCAGAAACGCCATAGCACTTAAAGGTTGAAAAAAATGT

TGAGTTGTAGAGCACAGAGTAATCAGCAACACAATTAGAAATTTTTTTTC

TCTCCCATGCATAGACAGAAGGGAATTTAGTAGCATTAAAAACCTCTCCA

AAAGGACACAAGTTTGTAATATTAGGGAATCTCACAACATCTCCTGAGGG

AACAACCCTGAAATTAGAGGTCTGGTAAATTCCTTTGTCAATCTCAAAGC

TCTTAACAGAGCATTTGAGTTCAGCAAGTGGATTTTGAGAACAATCAACA

GCCTCTGTGATTGTACCATTTTCATCATACTTGAGCATAAATGTAGTTGG

CTTTAAATAGCCAACAAAATAGGCTGCAGCTGACGTGCCCCAAATGTCTT

GAGCAGGTGAAAAGGCTGTAAGAATGGCTCTAAAATTTGTAATGTTAATA

CCAAGAGGCAACTTAAAAATAGGTTTCAAAGTGTTAAAACCAGAAGGTAG

ATCACGAACTACATCTATAGGTTGATAGCCCTTATAAACATAGAGAAACC

CATCTTTATTTTTAAAAACAAACTCTCGTAAGTGTTTAAAATTACCTGAC

TTTTCTGAAACATCAAGCGAAAAGGCATCAGATATGTACTCGAAAGTGCA

ATTAAATGCATTATCGAATATCATAGTATGTGTCTGTGTACCCATGGGTT

TAGAAACAGCAAAGAAAGGGTTGTCACACAATTCAAAGTTACATGCTCGT

ATAACAACATTAGTAGAATTGTTAATAATAATCACCGACTGTGACTTGTT

GTTCATGGTAGAACCAAAAACCCAACCACGGACAACATTTGATTTCTCTG

TGGCAGCAAAATAAATACCATCCTTAAAAGGTATGACAGGGTTGCCAAAC

GTATGATTAATAGTATGAAACCCTGTAACATTAGAATAAAATGGAAGAAA

TAAATCCTGAGTTAAATAAAGAGTGTCTGATCTAAAAATTTCATCAGGAT

AGTAAACCCCCCTCATAGATGAAGTATGTTGAGTGTAATTAGGAGCTTGA

ACATCATCAAAAGTGGTGCACCGGTCAAGGTCACTACCACTAGTGAGAGT

AAGAAATAATAAGAAAATAAACATGTTCGTTTAGAGAACAGATCTACAAG

AGATCGAGGTTGGTTGGCTTTTCCTGGGTAGGTAAAA

>Contig229

TATTGGGGCCTTCAAGAATTGGGAAAATATGAGCAATATATTAAATGGCC

TTGGTATGTTTGGCTCGGCTTCATTGCTGGACTAATTGCCATCGTCATGG

TTACAATCTTGCTTTGTTGCATGACTAGTTGTCGCAGTTGCCTCAAGGGT

GCATGCTCTTGTGGTTCTTGCTGCAAGTTTGATGAGGATGACTCTGAGCC

AGTTCTCAAGGGTGTCAAATTACATTACACATAAACGAACTTATGGATTT

GTTTATGAGATTTTTTACTCTTGGATCAATTACTGCACAGCCAGTAAAAA

TTGACAATGCTTCTCCTGCAAGTACTGTTCATGCTACAGCAACGATACCG

CTACAAGCCTCACTCCCTTTCGGATGGCTTGTTATTGGCGTTGCATTTCT

TGCTGTTTTTCAGAGCGCTACCAAAATAATTGCGCTCAATAAAAGATGGC

AGCTAGCCCTTTATAAGGGCTTCCAGTTCATTTGCAATTTACTGCTGCTA

TTTGTTACCATCTATTCACATCTTTTGCTTGTCGCTGCAGGTATGGAGGC

GCAATTTTTGTACCTCTATGCCTTGATATATTTTCTACAATGCATCAACG

CATGTAGAATTATTATGAGATGTTGGCTTTGTTGGAAGTGCAAATCCAAG

AACCCATTACTTTATGATGCCAACTACTTTGTTTGCTGGCACACACATAA

CTATGACTACTGTATACCATATAACAGTGTCACAGATACAATTGTCGTTA

CTGAAGGTGACGGCATTTCAACACCAAAACTCAAAGAAGACTACCAAATT

GGTGGTTATTCTGAGGATAGGCACTCAGGTGTTAAAGACTATGTCGTTGT

ACATGGCTATTTCACCGAAGTTTACTACCAGCTTGAGTCTACACAAATTA

CTACAGACACTGGTATTGAAAATGCTACATTCTTCATCTTTAACAAGCTT

GTTAAAGACCCACCGAATGTGCAAATACACACAATCGACGGCTCTTCAGG

AGTTGCTAATCCAGCAATGGATCCAATTTATGATGAGCCGACGACGACTA

CTAGCGTGCCTTTGTAAGCACAAGAAAGTGAGTACGAACTTATGTACTCA

TTCGTTTCGGAAGAAACAGGTACGTTAATAGTTAATAGCGTACTTCTTTT

TCTTGCTTTCGTGGTATTCTTGCTAGTCACACTAGCCATCCTTACTGCGC

TTCGATTGTGTGCGTACTGCTGCAATATTGTTAACGTGAGTTTAGTAAAA

CCAACGGTTTACGTCTACTCGCGTGTTAAAAATCTGAACTCTTCTGAAGG

AGTTCCTGATCTTCTGGTCTAAACGAACTAACTATTATTATTATTCTGTT

TGGAACTTTAACATTGCTTATCATGGCAGACAACGGTACTATTACCGTTG

AGGAGCTTAAACAACTCCTGGAACAATGGAACCTAGTAATAGGTTTCCTA

TTCCTAGCCTGGATTATGTTACTACAATTTGCCTATTCTAATCGGAACAG

GTTTTTGTACATAATAAAGCTTGTTTTCCTCTGGCTCTTGTGGCCAGTAA

CACTTGCTTGTTTTGTGCTTGCTGCTGTCTACAGAATTAATTGGGTGACT

GGCGGGATTGCGATTGCAATGGCTTGTATTGTAGGCTTGATGTGGCTTAG

CTACTTCGTTGCTTCCTTCAGGCTGTTTGCTCGTACCCGCTCAATGTGGT

CATTCAACCCAGAAACAAACATTCTTCTCAATGTGCCTCTCCGGGGGACA

ATTGTGACCAGACCGCTCATGGAAAGTGAACTTGTCATTGGTGCTGTGAT

CATTCGTGGTCACTTGCGAATGGCCGGACACCCCCTAGGGCGCTGTGACA

TTAAGGACCTGCCAAAAGAGATCACTGTGGCTACATCACGAACGCTTTCT

TATTACAAATTAGGAGCGTCGCAGCGTGTAGGCACTGATTCAGGTTTTGC

TGCATACAACCGCTACCGTATTGGAAACTATAAATTAAATACAGACCACG

CCGGTAGCAACGACAATATTGCTTTGCTAGTACAGTAAGTGACAACAGAT

GTTTCATCTTGTTGACTTCCAGGTTACAATAGCAGAGATATTGATTATCA

TTATGAGGACTTTCAGGATTGCTATTTGGAATCTTGACGTTATAATAAGT

TCAATAGTGAGACAATTATTTAAGCCTCTAACTAAGAAGAATTATTCGGA

GTTAGATGATGAAGAACCTATGGAGTTAGATTATCCATAAAACGAACATG

AAAATTATTCTCTTCCTGACATTGATTGTATTTACATCTTGCGAGCTATA

TCACTATCAGGAGTGTGTTAGAGGTACGACTGTACTACTAAAAGAACCTT

GCCCATCAGGAACATACGAGGGCAATTCACCATTTCACCCTCTTGCTGAC

AATAAATTTGCACTAACTTGCACTAGCACACACTTTGCTTTTGCTTGTGC

TGACGGTACTCGACATACCTATCAGCTGCGTGCAAGATCAGTTTCACCAA

AACTTTTCATCAGACAAGAGGAGGTTCAACAAGAGCTCTACTCGCCACTT

TTTCTCATTGTTGCTGCTCTAGTATTTTTAATACTTTGCTTCACCATTAA

GAGAAAGACAGAATGAATGAGCTCACTTTAATTGACTTCTATTTGTGCTT

TTTAGCCTTTCTGCTATTCCTTGTTTTAATAATGCTTATTATATTTTGGT

TTTCACTCGAAATCCAGGATCTAGAAGAACCTTGTACCAAAGTCTAAACG

AACATGAAACTTCTCATTGTTTTGACTTGTATTTCTCTATGCAGTTGCAT

ATGCACTGTAGTACAGCGCTGTGCATCTAATAAACCTCATGTGCTTGAAG

ATCCTTGTAAGGTACAACACTAGGGGTAATACTTATAGCACTGCTTGGCT

TTGTGCTCTAGGAAAGGTTTTACCTTTTCATAGATGGCACACTATGGTTC

AAACATGCACACCTAATGTTACTATCAACTGTCAAGATCCAGCTGGTGGT

GCGCTTATAGCTAGGTGTTGGTACCTTCATGAAGGTCACCAAACTGCTGC

ATTTAGAGACGTACTTGTTGTTTTAAATAAACGAACAAATTAAAATGTCT

GATAATGGACCCCAATCAAACCAACGTAGTGCCCCCCGCATTACATTTGG

TGGACCCACAGATTCAACTGACAATAACCAGAATGGAGGACGCAATGGGG

CAAGGCCAAAACAGCGCCGACCCCAAGGTTTACCCAATAATACTGCGTCT

TGGTTCACAGCTCTCACTCAGCATGGCAAGGAGGAACTTAGATTCCCTCG

AGGCCAGGGCGTTCCAATCAACACCAATAGTGGTCCAGATGACCAAATTG

GCTACTACCGAAGAGCTACCCGACGAGTTCGTGGTGGTGACGGCAAAATG

AAAGAGCTCAGCCCCAGATGGTACTTCTATTACCTAGGAACTGGCCCAGA

AGCTTCACTTCCCTACGGCGCTAACAAAGAAGGCATCGTATGGGTTGCAA

CTGAGGGAGCCTTGAATACACCCAAAGACCACATTGGCACCCGCAATCCT

AATAACAATGCTGCCACCGTGCTACAACTTCCTCAAGGAACAACATTGCC

AAAAGGCTTCTACGCAGAGGGAAGCAGAGGCGGCAGTCAAGCCTCTTCTC

GCTCCTCATCACGTAGTCGCGGTAATTCAAGAAATTCAACTCCTGGCAGC

AGTAGGGGAAATTCTCCTGCTCGAATGGCTAGCGGAGGTGGTGAAACTGC

CCTCGCGCTATTGCTGCTAGACAGATTGAACCAGCTTGAGAGCAAAGTTT

CTGGTAAAGGCCAACAACAACAAGGCCAAACTGTCACTAAGAAATCTGCT

GCTGAGGCATCTAAAAAGCCTCGCCAAAAACGTACTGCCACAAAACAGTA

CAACGTCACTCAAGCATTTGGGAGACGTGGTCCAGAACAAACCCAAGGAA

ATTTCGGGGACCAAGACCTAATCAGACAAGGAACTGATTACAAACATTGG

CCGCAAATTGCACAATTTGCTCCAAGTGCCTCTGCATTCTTTGGAATGTC

ACGCATTGGCATGGAAGTCACACCTTCGGGAACATGGCTGACTTATCATG

GAGCCATTAAATTGGATGACAAAGATCCACAATTCAAAGACAACGTCATA

CTGCTGAACAAGCACATTGACGCATACAAAACATTCCCACCAACAGAGCC

TAAAAAGGACAAAAAGAAAAAGACTGATGAAGCTCAGCCTTTGCCGCAGA

GACAAAAGAAGCAGCCCACTGTGACTCTTCTTCCTGCGGCTGACATGGAT

GATTTCTCCAGACAACTTCAAAATTCCATGAGTGGAGCTTCTGCTGATTC

AACTCAGGCATAAACACTCATGATGACCACACAAGGCAGATGGGCTATGT

AAACGTTTTCGCAATTCCGTTTACGATACATAGTCTACTCTTGTGCAGAA

TGAATTCTCGTAACTAAACAGCACAAGTAGGTTTAGTTAACTTTAATCTC

ACATAGCAATCTTTAATCAATGTGTAACATTAGGGAGGACTTGAAAGAGC

CACCACATTTTCATCGAGGCCACGCGGAGTACGATCGAGGGTACAGTGAA

TAATGCTAGGGAGAGCTGCCTATATGGAAGAGCCCTAATGTGTAAAATTA

ATTTTAGTAGTGCTATCCCCATGTGATTTTAATAGCTTCTTAGGAGAAAT

A

>Contig246

CCCTCGATAGGGCTTCCTCAAGTGTATAACCAGCACATCCTTGTCCAGGG

TACGTGGTTATATACTCATCATCTGGCACTTTCTTCAAAGCTCTTGAGAG

CATCTCAGTAGTGCCACCAGCCTTTTTGGAGGGTATTACAACACAAGTGA

TATCACCACTAGTGATAACATCACCTACCATGTAAGGTGCATCCTTCTCA

AGGAAAGACATATCTTCACCTCTAAGCATGTTCTGAGAATCATGGTAAAG

CTTACCATTGATATCAGCAAACAAGAGTAACTTATTGGTAAGAAACTTAG

TTTCTTCCAGTGTTGTGGTAACCTCATCAATGCAGGCCTTAATTTTTGGC

TTCACATCGACAGGCTTCTGTACGACAGATTTCTCCTCAGTTTTGGAATC

TTCTGTGTTTGGTGGCTCCTCTTGTTTAGGTGCTTCCACTCTAGGCTTCA

GGTTATCAAGATAATCCATGACAACCTGCTCATAAAGAGCTTTGTCATTG

ACTGCAATATAAACCTGTGTACGAACCGTCTGCACGCACACTTGTAAAGA

CTGAAGTGGTTTAGCACCAAATATGCCTGCTGACAACAATGGTGCAAGTA

AGATGTCCTGTGAATTGAAATTTTCATATGCTGCCTTAAGAAGCTGGATG

TCCTCACCTGCATTTAGGTTAGGTCCAACAACATGCAGACACTTCTTAGC

AAGATTATGTCCAGAAAGCAAACAAGACCCTCCTACTGTAAGAGGGCCAT

TTAGCTTAATGTAATCATCACTCTCCTTTTGCATGGCACCATTGGTTGCC

TTGTTGAGTGCACCTGCTACACCACCACCATGTTTCAGGTGTATGTTAGC

AGCATTTACAATCACCATAGGATTAGCACTTTGTGCCTCCTTAACGATGT

CAACACATTTAATGGCAACATTGTCAGTAAGTTTTAAATAACCAGTAAAC

TGATTAACTGGTTCTTCAGGTGTAGGTTCTGGTTCTGGCTCAATCTCTGA

TTGCTCAGTAGTATCATCCAGCCAGTCTTCCTCTTCTTCTTCCTCAACTC

GAACTGTTTCAGCTGAGGCACCAAATTCCAGAGGGAGACCTTGATAATCA

TCCTCTGTACCGTACTCATGTTCACAGGTTTCATCAATTTCTTCTTCCTC

ACACTCTGCATCGTCCTCTTCTTCCTCATCTGGAGGGTAAAAGGAACAAT

ACATACGTGATGAAAAGTTTTCTTCACCAGCATCATCAAATAAGTAGAAT

GTAGCTACACTCCACTCATCAAGATCAATACCCATGTTGGTAAGGAGATC

AGAAACTGGTTGTAAAGTCTTCACAACAGCCTCTGCTACAACACATGCAA

ACTCAGTAACTTCGGTACCGGATTCAACAGTGTAGACAGAGCACTTTTCA

TTAAGCACTTTGTCAACACGTTCATCAAGCTCAAATGTGATTCTCACATT

CTTGTAACCTTGAACTTCCCAAACAGTATCTTCTCCAAAGGTTACACCTT

TAATTGGTGCACCCCCTTTTAAGCGAAAGACATTGTTTGTAGCCAGTAAA

CCAGGAGACAATGCGCAGTATTGTTCTTTGTCCTTAATCTCTAAGAGCAT

GAGGCCATTTACACAGACTGGTGTGCCAACGATAGCTCCATTTGTGAAGC

TATCAACGGGCGTCTCGAGTGCTTCGAGTTCACCGTTCTTGAGAACAACC

TCCTCAGAGGTAAGTACTGTGTCATGTGAATCACCTTCAAGAAAGGTTAC

TTCTTTTGGTGCCTTAAGAGGCATGAGTAGTTGCAGCTGCTCCTTGCCAC

GTATACACTGACGGTAAAGTCCCTTGCTTTGAGCGATGAAGACTTCACCT

AAGTTGAGTGATCGCAACTTTGCGCCAGCGATAGTGACTTGATCAATGCA

CATTTCGAGTGCCTTGTTAACAACATCAATGAAGCATTTTACACAATCCT

TGATGTTATCTGAAGCAACCTGTATTTGACCCTTGACGATGTCAAAAACA

CCTGTAATGAGAAATTTGAGAATCTCCCAAGCATCCTTGAGAAATTCAAC

TCCTGCACTAAGTTTCGCCTCAATCCATTCAAAGATAGGCCTGAGTTTTT

CAACAGTAGTGCCCAAAAGATTAGACAACCACTGAGAAGTCTGTTGTACA

AGACCACCAGTTACATATGCCATAATAATGACACTGTTGGTGAGCAGGTC

TGAAGTATAAACCATGGCGTCGACAAGACGTAATGACTGTTCAGAAATAC

CATCAAGTATGGTGACAGCTGCTCTTTGCAAATCAGGAATTGAGTGGTTT

GCTGCATCAAGTGTGCGCGCAAAAATTGATCTGATAACACCAGCAGCCTG

TGAGGGAAAACCACACAGTGGTGTTAAAACTGATCTCTGTTGTCCAATGT

TCCAAGCACCTTTTACGGGCTTTCCCTTGGTAACTTTATAGTTACCGCAG

GACTCAACAATGGTTTTGAAAGACTTGTAATCAAGACTCTTTATAGTGTC

AATAAAGGCACTTGTAGAAGCAGAGAAAGATGCCAAAATGATGGCAACCT

CTTCATTCAAATGAAAATCGCCAACAATGTTAATGTTAACACGTTCACGA

CTCAGTATCTCAAGGAGATCCTCATTCAAGGTCTCCACATTGTCACCAGT

AATGCCAGTATGGCCTGAGCCAATATCAGCACTAGCACGAGGAACCCAGT

AGGCACGCTTATTATAGCAGCCAACATAGGCAAACACACAGCCTCCAAAA

CATCTAGTCCTACCTCCCTTGCGGAGTCGAGTTTCAATGTTTGAGTGGTT

GTGATAATCTGCAACACTATGCTCAGGTCCAATCTCTGGGTCTTGACAGG

CAGGACATGGCATTTTCACTACAGCATTAGTAGGTAGGTACCCACATGTA

GTAGGTCCTTCAATAACTAAATTTTCAGTGCCACAATGTTCACAAGTGGC

TTTCAGAAAGTCGCACGTCTGCCATGAAACTTCATCGCAATGATTACATT

TCATCAAGGTAGACAAGTGCATATTGTTACACTCCTGTGGAGATGCAACA

GGGTACACAGAGCGTATACGCCCCATGAAACCCTCAGTCTTTTTCTTTTC

AACACGTGGTTGAATGACTTTGACTTTTGAGTTAAGAGGAAACACAAACT

TTGGGCATTCCCCTTTGAAAGTGTCAAATTTCTTGGCACTCTTAATTTCG

AAGGGTGTCTGGTGCTCGTAGCTCTTATCAGAGCGCTCAGTGAACCAGGC

AATTTCATGCTCATGGTCACGGCAGCAGTAGACACCTCTCTTCGACTCGA

TGTAATCAAGTTGTTCGGAAAGAGTGCACATTGACTTGCCCGCGCGTGCG

AGAAAATCTTTGATGCAATCAAGAGGGTACCCATCTGGGCCACAGAAATT

GTTGTCGACATAGCGAGTGACTGCACCTCCATTGAGCTCACGAAAAAGTT

CACGGAGTGCACCACTGCCATGCTTAGTGTTCCAGTTTTGTTCATAATCT

TCAATGGGATCAGTGCCAAGCTCGTCACCTAAGTCATAAGACTTTAGATC

GATGCCATAGCTATGACCACCGGCTCCCTTATTACCGTTCTTACGAAGAA

GAACATTGCGGTATGCAATTGGGGTTTCGCCCACATGTGGCACGAGTACT

CCCAGTGTTATACCGCTACGACCGTACTGAATGCCGTCCATTTCTGCAAC

CAGCTCAACGACCTTGTGGCCGTGATTGGTGCTTAAGGCATCAGAACGTT

TAATGAACACATAGGGCTGTTCAAGCTGGGGCAGTACGCCTTTTTCCAGC

TCTACTAGACCACAAGTGCCATTTTTGAGGTGTTCACGTGCCTCCGATAG

GGCCTCTTCCACAGAGTCCCCGAAGCCACGCACTAGCACGTCTCTAACCT

GAAGGACAGGCAAACTGAGTTGGACGTGTGTTTTCTCGTTGACACCAAGA

ACAAGGCTCTCCATCTTACCTTTCGGTCACACCCGGACGAAACCTAGGTA

TGCTGATGATCGACTGCAACACGGACGAAACCGTAAGCAGTCTGCAGAAG

AGGGACGAGTTACTCGTTTCTTGTCAACGACAGTAAAATTTATTATTGTT

TATACTGCGTAGGTGCACTAGGCATGCAGCCGAGCGACAGCTACACAGAT

TTTAAAGTTCGTTTAGAGAACAGATCTACAAGAGATCGAGGTTGGTTGGC

TTTTCCTGGGTAGGTAAAAACCTAATA
