# Supplementary material for: Viral Discovery and Sequence Recovery Using DNA Microarrays
Source: PLoS Biol. 2003 Nov 17;1(2):e2. doi: 10.1371/journal.pbio.0000002 (PMC261870; doi:10.1371/journal.pbio.0000002)
Supplement: Protocol S1 — (28 KB DOC) [file pbio.0000002.sd002.doc]

# Round A/B/C Random Amplification Protocol

Supplement to

D. Wang et al. “Viral Discovery and Sequence Recovery Using DNA Microarrays”

DeRisi Laboratory, University of California San Francisco

Adapted from Bohlander et al. *Genomics* **13** (1992) 1322.

************************************************************************

Take standard PCR precautions. Use filter tips, UV irradiation boxes and separate areas for sample setup and product analysis. Wear gloves and be careful of contamination as any nucleic acids can be amplified by this protocol. Always run a negative control sample with water only (no template) to make sure the reagents are not contaminated with nucleic acids.

************************************************************************

This protocol consists of three ‘Rounds’ of enzymatic reactions. In Round A, reverse transcriptase is used for 2 cycles of 1st strand cDNA synthesis with PrimerA. Sequenase is used for 2nd strand synthesis. During Round B, the specific primer B is used to amplify the templates previously generated. Finally, Round C consists of additional PCR cycles to incorporate either amino allyl dUTP or Cy-dye-coupled nucleotide.

Necessary Reagents:

Round A

Reverse Transcriptase

10X Reverse Transcription Buffer

25 mM dNTP mix

0.1 M DTT

40 pmol/ul Primer A: GTT TCC CAG TCA CGA TCN NNN NNN NN

Sequenase(13 units/ul) US Biochemical cat# 70775

5X Sequenase Buffer

Sequenase Dilution Buffer

Round B

10X PCR Buffer (500 mM KCl, 100 mM Tris pH 8.3)

50 mM MgCl2

25 mM dNTP mix

5 unit/ul Taq polymerase

100 pmol/ul Primer B: GTT TCC CAG TCA CGA TC

Round C

100X modified dNTP mix

25 mM dATP

25 mM dCTP

25 mM dGTP

10 mM dTTP

15 mM aminoallyl-dUTP or Cy-dUTP

(The ratio of aa-dUTP to dTTP can be altered/optimized)

# ROUND A

# 1st strand synthesis with RT

Always include negative control (H­20 only) and positive control (we use 50 ng of HeLa cell total RNA) templates.

In 500 uL tube, mix RNA with 1 ul primer A (40 pmol/ul) to a final volume of 10 ul.

Heat to 65 °C, 5 min.

Cool at room temp 5 min.

Add 10 uL of 2X enzyme mix

Make master mix of:

2X enzyme mix

2.0 ul 10X RT Buffer

0.4 ul 25 mM dNTP mix (final concentration 500 uM each nucleotide)

3.6 ul H2O

2.0 ul 0.1M DTT

2.0 ul Reverse Transcriptase (Stratascript)

Incubate at 42°C, 30 min.

Heat to 65°C 5 min

Cool at Room Temp 5 min

Add 1 uL RT

Incubate additional 42°C, 30 min.

**2nd strand synthesis with Sequenase**

Heat sample to 94°C 2 min. Rapidly cool to 10°C and hold at 10°C 5 min.

Add 10 uL Sequenase mix for a total RXN volume of 30 uL.

Sequenase Mix

2.0 ul 5X Sequenase Buffer

7.7 ul H­2O

0.3 ul Sequenase

Ramp from 10°C to 37°C over 8 min.

Hold at 37°C for 8 min; rapid ramp to 94°C and hold for 2min

Rapid ramp to 10°C and hold for 5 min at 10°C while adding 1.2 ul of diluted Sequenase (1:4 dilution)

Ramp from 10°C to 37°C over 8 min.

Hold at 37°C for 8 min, ramp to 94°C and hold 8 min.

**ROUND B**

Round A Template 6

50 mM MgCl2 4

10X PCR Buffer 10

25 mM dNTP 1

Primer B (100pmol/ul) 1

Taq Polymerase (hot start) 1

Water 77

Round B Cycles:

30 sec 94°C

30 sec 40°C

30 sec 50°C

1 min 72°C

Run 40 cycles (or less), depending on the amount of starting material.

Run 10 uL on 1% agarose gel. A smear of DNA should be present between 500bp –1kb. It may be necessary to remove aliquots every 2 cycles to check the amplification in order to optimize the number of cycles. It is best to use the minimal number of cycles that generates a visible shmear. Make sure there is no DNA in the negative control lane!

# ROUND C

Use 10 uL of Round B to seed the Round C reaction:

Round B Template 10

50 mM MgCl2 4

10X PCR Buffer 10

100X modified dNTP mix 1

Primer B(100pmol/ul) 1

Taq 1

Water 73

30 sec 94°C

30 sec 40°C

30 sec 50°C

1 min 72°C

20 cycles
