# Supplementary material for: Viral Discovery and Sequence Recovery Using DNA Microarrays
Source: PLoS Biol. 2003 Nov 17;1(2):e2. doi: 10.1371/journal.pbio.0000002 (PMC261870; doi:10.1371/journal.pbio.0000002)
Supplement: Protocol S2 — (39.5 KB DOC) [file pbio.0000002.sd003.doc]

### Microarray Sequence Recovery Protocol

Supplement to

D. Wang et al. “Viral Discovery and Sequence Recovery Using DNA Microarrays”

DeRisi Laboratory, University of California San Francisco

This protocol describes the recovery of hybridized material from spotted oligonucleotide glass microarrays. In our laboratory, we typically recover material that has been amplified using the Round A/B/C Random Amplification Protocol (see accompanying protocol). Thus DNA molecules hybridized to the microarray possess defined sequences at each end (Primer B) that can be used to further amplify the material following recovery from the microarray.

### Protocol

1. Clean a tungsten wire probe (Omega Engineering, Inc.) with 2% bleach and rinse with copious amounts of water. Snap dry on a flame or wipe dry with a clean tissue.

2. Visualize spots on the microarray by fluorescence microscopy using Cy3 or Cy5 excitation wavelength.

4. Mount the cleaned probe on a micromanipulator (Technical Instrument San Francisco, Inc.) proximal to the microscope such that the probe is just above and at ~ 30 deg angle to the microarray surface.

5. Lower the probe on a spot of interest and drag it across the spot area 5 to 10 times.

6. Raise the probe away from the microarray surface and dip the probe tip directly into a PCR tube with 100 ul PCR mix (see below) to seed the reaction.

7. Repeat steps 1 – 7 for recovery from additional spots.

8. PCR amplify recovered material using 40 cycles of [30 sec at 94°C, 30 sec at 40°C, 30 sec at 50°C, and 1 min at 72°C].

# PCR Mix

50 mM MgCl_2_ 4

10X PCR Buffer 10

25 mM dNTP 1

Primer B (100pmol/ul) 1

Taq Polymerase 1

Water 83

8. Subsequent to PCR amplification, material is cloned using TOPO-TA Cloning kit (Invitrogen) following manufacturer’s instructions.

9. Miniprep and sequence plasmids.

### Alternative Protocol

70mers of interest from the original hybridization can be manually spotted onto blank microarray slides, and the locations of each 70mer can be identified by physically marking the obverse side of the slide with a diamond pen. The hybridization can then be repeated and spots can be scraped as described above, except there is no need for spot visualization using microscopy.
