# Supplementary material for: Viral Discovery and Sequence Recovery Using DNA Microarrays
Source: PLoS Biol. 2003 Nov 17;1(2):e2. doi: 10.1371/journal.pbio.0000002 (PMC261870; doi:10.1371/journal.pbio.0000002)
Supplement: Table S1 — (97 KB DOC) [file pbio.0000002.st001.doc]

| OligoID | Virus | Sequence | BLAST* Identities |
| --- | --- | --- | --- |
| 9635576_275 | Turkey astro | GATGCTTGAGAAAAATTGATGCCGAGGCCACGCCGGGTAGGATCGAGGGTACAGCATCGGTGCACTAACT | 29/32 |
| 9635576_273 | Turkey astro | AGGCATCTTGAGGAGGAACTCCAGTTTTGGAAATCTAAAGCAACACAGCTGATGCTTGAGAAAAATTGAT | No Hit |
| 9635576_265 | Turkey astro | ACTGATGTTCAAGCAGAACCACTAGCCTTATGACCTTATATAATGTGGATGCCGGTAGAGCACCTATCAC | No Hit |
| 9635576_172 | Turkey astro | CTGGACAATTTTCTACCACAGTTGACAACAACTTTGTTAATGTTTGGTTAACTGTCTTTGAGTTAGCTTA | No Hit |
| 9635576_163 | Turkey astro | ATGGTGAACATAGGTACTATGTCGAACTTGATTGGACTAGATTTGATGGCACCATACCACCAGAATTGTT | No Hit |
| 9635572_255 | Ovine astro | ATCACTTCAATCCCGAGGCCACGCCGAGTAGGATCGAGGGTACAGGATTGTTTGATTTTTTAATCAATTA | 30/32 |
| 9635572_251 | Ovine astro | ATGACGATGATTTTGAATGCCTGCGTAGTACGCCCTTACAGCAAATCTATGAGGGCGTACGCGGCCTTCG | No Hit |
| 9635572_247 | Ovine astro | ACCTCGGACGATGAGCTTGTTGCACGCATTCTTGGGCAACTCCAGACCCGGCTTAAATTTGCCGCTGGGT | No Hit |
| 9635572_204 | Ovine astro | CGTGGCCCCCGCACCCTTTGGGTGGCTCATAAAAGGCGGCTGGTGGTTTGTGAAGAAAATACTTGGTAGA | No Hit |
| 9635572_149 | Ovine astro | AGTTGTTGCCATGTACAAGGACATTTTTGGCATGTGGGTGAAACCTGAGAAAGTCAAGGTTTCTCACTCC | No Hit |
| 9630726_73 | Human astro | CACTGGAGGTGCTGTCATAATAGACCCAGCAGACTTCCATCCAGTGAAAGCCCCATCTCAGGTGGAATTG | No Hit |
| 9630726_269 | Human astro | TGCACATCTGGAAGCCGCGGCCACGCCGAGTAGGAACGAGGGTACAGCTTCCTTCTTTTCTGTCTCTGTT | 26/29 |
| 9630726_173 | Human astro | ACTCTGGCCTTCCAGCCAGACTCACAGAAGAGCAACTCCATCGCATTTGGAGGGGAGGACCAAAGAAGTG | No Hit |
| 9630726_147 | Human astro | TATACCACCAGCACTCTTTAAACACATCAAAGAAATTAGGTGGAATTTCATCAATAAAGACCAACGTGAA | No Hit |
| 9630726_114 | Human astro | AAAACAAGGCCCCAAAAAACTACAAAGGGCCCCAGAAGACCAAGGGGCCCAAAACTACCACTCATTAGAT | No Hit |
| 9629812_776 | Murine hepatitis | CCTGGCTGTAATGGTGGCAGTTTGTATGTTAACAAACATGCATTCCACACCAGTCCCTTTACCCGGGCTG | 42/47 |
| 9629812_772 | Murine hepatitis | AGATGGTTTGTGTATGTTTTGGAACTGCAATGTGGATAAGTATCCAGCGAATGCAGTTGTGTGTAGGTTT | 25/27 |
| 9629812_639 | Murine hepatitis | AGGCCAAATGTTGGGTAGAAACAGACATCGAAAAGGGACCGCATGAATTTTGTTCTCAACATACAATGCT | 30/35 |
| 9629812_617 | Murine hepatitis | GTACTCATGGGTTGGGACTATCCTAAATGTGATCGTGCTATGCCAAACATACTGCGTATTGTTAGTAGTT | 24/26 |
| 9629812_616 | Murine hepatitis | CCTTATTAAAGATGTTGATAGTCCTGTACTCATGGGTTGGGACTATCCTAAATGTGATCGTGCTATGCCA | 24/26 |
| 9629812_603 | Murine hepatitis | ATAATTATGATAAGAGTGCTGGCTATCCATTTAATAAATTTGGAAAGGCCAGGCTCTATTATGAGGCATT | No Hit |
| 9629812_535 | Murine hepatitis | CCACTAATCAGGATTCTTATGGTGGTGCTTCCGTTTGTATATATTGCCGCTCGCGTGTTGAACATCCAGA | No Hit |
| 9626535_727 | Avian infectious bronchitis | CAGGCTGTAATGGTGGTAGTCTGTACGTTAACAAACATGCATTCTACACACCTAAATTTGACCGCATTAG | 42/49 |
| 9626535_575 | Avian infectious bronchitis | TATTTATGTTAAACCTGGTGGCACTAGCAGTGGTGATGCTACTACTGCTTATGCAAACAGTGTTTTTAAC | 35/39 |
| 9626535_574 | Avian infectious bronchitis | CTGAAACTGTACTTGCTACAGGTGGTATTTATGTTAAACCTGGTGGCACTAGCAGTGGTGATGCTACTAC | No Hit |
| 9626535_568 | Avian infectious bronchitis | TTATGGGTTGGGATTATCCTAAGTGTGATAGAGCAATGCCTAATTTGTTGCGTATAGCAGCATCCTTAGT | 39/43 |
| 9626535_564 | Avian infectious bronchitis | AACACTAGAAATGCTTCTGTAGTTATTGGAACAACCAAGTTTTATGGCGGTTGGGACAACATGTTGAGAA | No Hit |
| 9626535_554 | Avian infectious bronchitis | CAACTTAGATAAGAGTGCAGGCTATCCATTTAATAAGTTTGGAAAAGCCCGCCTCTATTATGAAATGAGT | No Hit |
| 9626535_547 | Avian infectious bronchitis | AACATTTTTTCTATCCTCAAACTGGTAATGCTGCTATAAACGATTATGATTATTATCGTTATAACAGGCC | 22/23 |
| 9626535_1099 | Avian infectious bronchitis | TAGAGTAGGTATAAAGATGCCAGTGCCGGGGCCACGCGGAGTACGATCGAGGGTACAGCACTAGGACGCC | 29/29 |
| 475891_157 | Human corona OC43 | GAGTATTATGTAAAATGGCCTTGGTATGTATGGCTTTTAATTGGCTTTGCTGGTGTAGCTATGCTTGTTT | 22/23 |
| 386388_98 | Human corona OC43 | ATATGGAGGAGTTTATTCAAACAAGCTCTCCTAAAGTTACTATTGATTGTGCTGCATTTGTCTGTGGTGA | No Hit |
| 386388_156 | Human corona OC43 | TCTCAAGGACATTGGTACATATGAATATTATGTAAAATGGCCTTGGTATGTATGGCTTTTAATCTGCCTT | 22/23 |
| 306155_156 | Human corona OC43 | TCAATCTCAAGGACATTGGTACATATGAATATTATGTAAAATGGCCTTGGTATGTATGGCTTTTAATCTG | 22/23 |
| 306155_137 | Human corona OC43 | ATAAATTTCTGTGGTAATGGTAATCATATTATATCATTAGTGCAGAATGCTCCATATGGTTTGTATTTTA | No Hit |
| 20514394_269 | Avian nephritis | ACTTTCCCGAGGCCACGGCGAGTAGCATCGAGGGTACAGGAAAGCTGGGACCATTGCATAGTCAACTAAT | 28/32 |
| 20514394_263 | Avian nephritis | CATCTCGAACGCGAAAGAGAAGACCTAATGAGGAGGCTCAGAGATCTAGACCTCCGGCGCTTTCAGATCT | No Hit |
| 20514394_261 | Avian nephritis | ACATCTCACTCGCGGGGTCGGTTATTGGCGACGAGTTTGATAGTGTGGATCATCTCGAACGCGAAAGAGA | No Hit |
| 20514394_160 | Avian nephritis | AAGAAAGGCAACCCATCTGGGCAATTTTCAACAACAGTTGACAACAACCTTGTTAATGAGTGGTTGACTG | No Hit |
| 20514394_152 | Avian nephritis | GCAGGACCCAAGTCCTAGAACTTGATTGGACGCGATTCGATGGCACCATTCCGGTTCAGCTCTTCCAAAG | No Hit |
| 19387576_736 | Porcine epidemic diarrhea | CTTGGAGGGTTGTAATGGTGGTTCACTGTATGTTAACAATCATGCATTCCACACACCGGCTTTTGACAAG | 32/37 |
| 19387576_732 | Porcine epidemic diarrhea | AATTTGATGGGTTGTGCTTGTTTTGGAATTGCAATGTGGACATGTATCCAGAATTTTCTGTGGTCTGTCG | No Hit |
| 19387576_700 | Porcine epidemic diarrhea | AAATGTCCCATTGCAATTAGGGTTTTCTAACGGTGTTGATTTTGTTGTCAGACCTGAAGGTTGCGTTGTA | No Hit |
| 19387576_566 | Porcine epidemic diarrhea | TATGAGTCTTTATCCTATGAGGAACAGGATGAACTTTATGCTTATACTAAGCGTAACATCCTGCCCACTA | 35/41 |
| 19387576_496 | Porcine epidemic diarrhea | GTACTAACCAGGATTCATATGGTGGTGCTTCCGTGTGTCTATATTGTAGAGCACATGTTGAGCATCCATC | No Hit |
| 15081544_766 | Bovine corona | CTGGCTGTAATGGAGGTAGTTTGTATGTTAATAAACATGCATTCCACACTAAACCCTTTTCTAGGGCAGC | 44/48 |
| 15081544_708 | Bovine corona | GGTTTGCAAACCCAGACCGTGGATTCTGCTCAAGGTTCTGAATATGATTATGTTATATATTCACAGACTG | 23/25 |
| 15081544_606 | Bovine corona | GATGTTGATAATCCTGTACTTATGGGTTGGGATTATCCTAAGTGTGATCGTGCTATGCCAAACATACTAC | 27/29 |
| 15081544_593 | Bovine corona | TAAGAGTGCTGGCTATCCATTTAATAAATTTGGAAAAGCCAGGCTCTATTATGAAGCATTATCATTTGAG | No Hit |
| 15081544_525 | Bovine corona | GGATTCATATGGTGGTGCGTCTGTTTGTATATATTGCCGCGCACGAGTTGAACACCCAGATGTTGATGGG | No Hit |
| 13399293_725 | Transmissible gastroenteritis | TTAGAAGGTTGTAATGGTGGTGCATTGTATGTTAATAACCATGCTTTCCACACACCAGCTTATGATAGAA | No Hit |
| 13399293_582 | Transmissible gastroenteritis | TTGAGTACTTTAGTTATTTGAGAAAACACTTTTCTATGATGATTTTATCTGATGATGGAGTTGTGTGCTA | 31/35 |
| 13399293_566 | Transmissible gastroenteritis | GATAATGGTTGTTTGATGGGATGGGACTATCCTAAGTGTGACCGTGCTTTACCTAATATGATTAGAATGG | No Hit |
| 13399293_552 | Transmissible gastroenteritis | TTGTTGTTACAAACTATGACAAGAGTGCTGGCTATCCTTTGAACAAATTTGGTAAAGCTAGACTTTACTA | No Hit |
| 13399293_517 | Transmissible gastroenteritis | AGGTGTTATAACACTTGACAACCAAGATCTTAATGGCAATTTCTACGATTTCGGCGATTTCGTGAAGACT | 33/38 |
| 13399293_485 | Transmissible gastroenteritis | CTCTTATGGTGGTGCTTCAGTTTGTATTTATTGCAGATGCCATGTTGAACATCCTGCTATTGATGGATTA | 31/36 |
| 12175745_728 | Human corona 229E | AAATGGATGGCTTGTGTTTGTTTTGGAATTGTAATGTGGATATGTACCCTGAATTCTCAATTGTTTGCAG | 25/27 |
| 12175745_675 | Human corona 229E | GCTTTTAGGCTTACAAACTCAGACAGTGGATTCTGCTCAAGGTAGTGAATATGACTATGTTATATTCGCA | 21/22 |
| 12175745_573 | Human corona 229E | CGATGTTGATGATCCTAAATTGATGGGATGGGACTATCCTAAGTGTGATAGAGCTATGCCCTCAATGATT | No Hit |
| 12175745_570 | Human corona 229E | CAGAAATGCCACCGTTGTTATCGGCACTACCAAGTTTTATGGCGGGTGGGATAATATGTTAAAGAACCTG | 29/33 |
| 12175745_501 | Human corona 229E | TTGATAACAGTTATTTAAACGAGTCCGGGGCTCTAGTGCCGCTCGACTAGAGCCCTGTAATGGTACAGAC | No Hit |
| 12175745_492 | Human corona 229E | ACACTACGCAGGACACATATGGTGGCGCGTCTGTTTGTATTTATTGCAGAGCACATGTTGCACATCCAAC | No Hit |

* BLASTn alignments to NC_004718 with e < 0.1
